# A phytic acid derived LiMn_0.5_Fe_0.5_PO_4_/Carbon composite of high energy density for lithium rechargeable batteries

**DOI:** 10.1038/s41598-019-43140-7

**Published:** 2019-04-30

**Authors:** Yan Meng, Yujue Wang, Zhaokun Zhang, Xiaojuan Chen, Yong Guo, Dan Xiao

**Affiliations:** 1 School of Chemical Engineering, Sichuan University, Chengdu, 610065 China; 2 Institute of New Energy and Low-Carbon Technology (INELT), Sichuan University, Chengdu, 610207 China; 3 College of Chemistry, Sichuan University, Chengdu, 610064 China

**Keywords:** Batteries, Batteries

## Abstract

A composite of olivine lithium manganese iron phosphate (LiMn_0.5_Fe_0.5_PO_4_), external carbon coating and internal embedded carbon flakes, EC-IC-LMFP, is prepared by using phytic acid (PhyA) as phosphorus source *via* solvothermal process followed by calcination. The composite with improved electronic conductivity and ion diffusivity presents an ultrahigh reversible specific capacity of 193 mAh g^−1^ at 0.1 C, and an excellent cycling stability of 93% capacity retention after 100 cycles at 1 C when applied as a cathode material for Li-ion batteries (LIBs). Additionally, the composite fine powders exhibit a special microstructure and its volumetric energy density is estimated to reach 1605 Wh L^−1^, much larger than the commercial LiFePO_4_.

## Introduction

The fast updating speed of computer, communication, and consumer (3C) products stimulates the development of rechargeable lithium-ion batteries (LIBs) with security, durability and high power/energy density^1^. Nowadays, layered LiCoO_2_ (LCO) covers the field of portable devices due to its high volumetric energy density (~2100 Wh L^−1^)^2^. Generally, the compact density of commercial LCO is 3.7 g mL^−1^, greater than the 2.3 g mL^−1^ of commercial LiFePO_4_ (LFP)^3^. Therefore, most of commercial lithium-ion batteries employ LCO as cathode material, for the compact density is the determinant of volumetric energy density^4,5^. The chemical/electrochemical stability of cathode materials is one of the factors for the safety of a cell. It is key to maintain the structural stability of Li_x_CoO_2_ in a limited compositional range of 0.5 < x < 1. Thereby it can only deliver an about 140 mAh g^−1^ specific capacity which is far below its theoretical value (275 mAh g^−1^)^6–8^. Considerable studies have been directed to the olivine-type LiMPO_4_ (M = Fe, Mn, Co, and Ni) compounds as one of the attractive candidates for high performance LIBs in that the orthorhombic structure shows excellent cycling and intrinsic stability^9–13^. Besides their high theoretical capacities (~170 mAhg^−1^), the strong covalent P–O bonds in the tetrahedral (PO_4_)^3−^ anion prohibits these materials from the oxygen release and increase the potential of the M^3+^/M^2+^ redox couple (>3.4 V vs. Li^+^/Li) as well as the cycle life^14–16^. Additionally, for large-scale Li-ion batteries, the olivine-type LiMPO_4_ is the most promising alternative to LiCoO_2_, which is thermally stable due to its low reactivity with electrolytes^17,18^ and non-existent heat evolution below 200 °C^19^. Therefore, batteries employing olivine LiMPO_4_ as cathode material has less risk of swelling, fire and explosion. Tailored nanometer-sized LFP particles with carbon coating have conquered the major obstacles of low ionic/electronic conductivity and approached the theoretical capacities at high rates^20^. Even so, the limit value of energy density of commercial LFP is just 500 Wh kg^−1^, which is less than that of LCO (520 Wh kg^−1^). It is worth noting that 2% graphene flakes were utilized to encase commercial LFP, where the modified-LFP exhibited a reversible capacity (208 mAh g^−1^) beyond the theoretical value and a high energy density (686 Wh kg^−1^). It was demonstrated that the few-layer graphene flakes can deliver a capacity higher than 2,000 mAh g^−1^, and the Li-ions in the electrolyte can reversibly react with the graphene flakes, bringing excess capacity^21^. Encouraged by the success of modified-LFP, many efforts had been devoted to prepare LFP with high energy density. A special phosphorus source, phytic acid (PhyA), was applied to synthesis LFP containing internal carbon flakes (IC). Combined with glucose-derived carbon (GC) coated, this composite displays a superb cycling stability and a large capacity of 192 mAhg^−1^ at 0.1 C^22^.

To further enhance the energy density, the isomorph LiMnPO_4_ (LMP) phase is quite attractive owing to a high redox potential (4.1 V vs. Li^+^/Li) of Mn^3+^/Mn^2+^ couple, which is still within the stability window of typical carbonate ester-based electrolytes^23,24^. Nevertheless, the LMP suffers sluggish kinetics of unusually poor electronic conductivity (<10^−12^ S cm^−1^) and ionic conductivity (<10^−16^ cm^2^ S^−1^), which are caused by Jahn-Teller anisotropic lattice distortion on the Mn^3+^ sites and the interface strain between the lithiated and delithiated phase^25,26^. Over the past two decades, these limitations of olivine LMP had been significantly overcome by a synergistic effect of Fe-doping and carbon coating. The phosphate-based LiMn_x_Fe_1−x_PO_4_ solid solution system possesses both advantages of LMP and LFP with relatively high operating voltage and high electrical conductivity^13^. Sun *et al*. reported a novel synthetic approach to prepare micrometer-sized LiMn_0.85_Fe_0.15_PO_4_ powders with high tap density (1.4 g cm^−3^). Consequently, the reference electrode displayed a high volumetric capacity (369 mAh cm^−3^), which is in fact of great importance since it determines the amount of the loaded active material^27^. Currently, Leng and her co-workers prepared a series of non-stoichiometric Li_x_Fe_0.2_Mn_0.8_PO_4_/C (x = 1.05, 1.1, 1.2) composite fibers by a simple electrospinning method. Following by annealing, the continuous and long Li_1.2_Fe_0.2_Mn_0.8_PO_4_/C fibers obtained and exhibited a reversible discharge capacity of 174 mAh g^−1^at 0.05 C^28^.

In this work, the LiMn_0.5_Fe_0.5_PO_4_ (LMFP) precursor with IC was synthesized by employed PhyA as both phosphorus and carbon source through a facile hydrothermal process. Later on, the glucose carbon source was introduced in following calcination to form an external coating (EC), and then the three-dimensional conducting network was built around LMFP. It needs to be emphasized that the as-obtained EC-IC-LMFP composite shows a high reversible discharge capacity of 193 mAh g^−1^ at 0.1 C and a higher average discharge voltage of 3.75 V than LFP (~3.4 V). Moreover, the fine powders present a special microstructure and the volumetric energy density is estimated to reach 1605 Wh L^−1^, about 24% less than that of commercial LCO (~2100 Wh L^−1^), and 28% larger than commercial LFP (~1250 Wh L^−1^). Combined with its long lifetime, eco-friendliness, lower cost and inherited safety feature, it will be a promising candidate to batteries for electrical vehicles and is possible to be an optional in the batteries for portable devices.

## Results and Discussion

**Figure 1:**
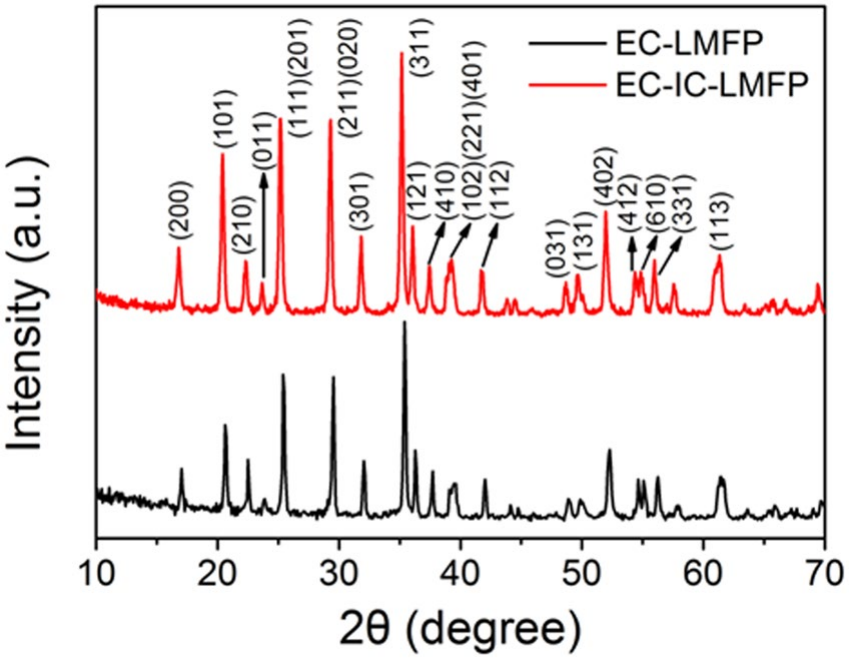
XRD patterns of EC-LMFP and EC-IC-LMFP.

**Figure 2:**
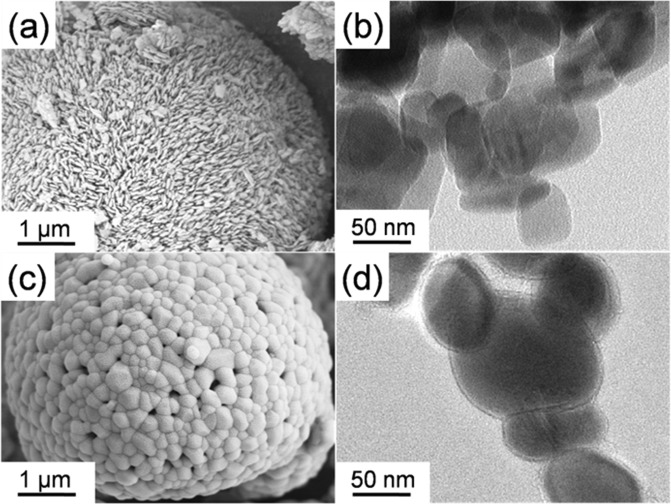
(**a**,**c**) SEM image of p-PhyA-LMFP and IC-LMFP; (**b**,**d**) TEM image of p-PhyA-LMFP and IC-LMFP.

**Figure 3:**
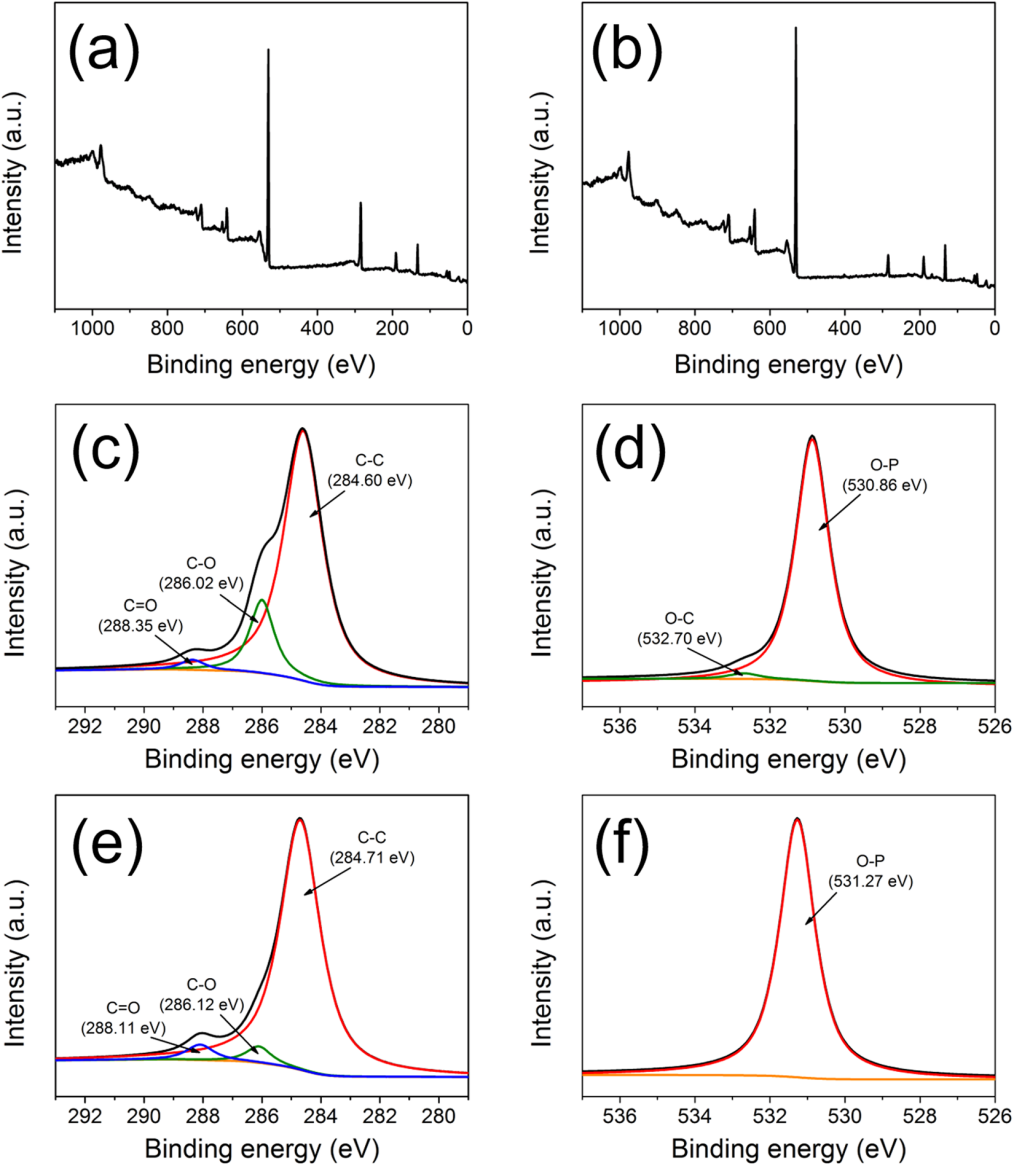
(**a**,**c**,**d**) XPS spectra of p-PhyA-LMFP: (**a**) survey spectrum, (**c**) C1s, (**d**) O1s; (**b**,**e**,**f**) XPS spectra of IC-LMFP: (**b**) survey spectrum, (**e**) C1s, (**f**) O1s.

X-ray diffraction (XRD) was first carried out to determine the crystal structure of EC-LMFP and EC-IC-LMFP (Fig. 1). It is clear that EC-LMFP and EC-IC-LMFP both correspond well to olivine LiMPO_4_, with no obvious impurity peaks detected, indicating the high purity of the products. In order to further confirm the phase composition of the as-prepared products, ICP-AES analysis was performed to obtain the relative contents of the elements in both EC-LMFP and EC-IC-LMFP, and the results are displayed in Table S1. It can be calculated that the molar ratio of Li:Mn:Fe:P is 1.03:0.49:0.53:1 for EC-LMFP and 1.29:0.50:0.51:1 for EC-IC-LMFP. Together with the XRD result, it can be confirmed that the products are both LiMn_0.5_Fe_0.5_PO_4_. Interestingly, EC-IC-LMFP shows an exceeded relative Li content, which is believed to provide additional capacity and discussed in the following context. Scanning electron microscopy (SEM) and transmission electron microscopy (TEM) were performed to characterize the morphology and structure of the precursor prepared using phytic acid (p-PhyA-LMFP) and IC-LMFP. From Fig. 2a,b, it can be clearly seen that the precursor is secondary particles stacked with large amounts of primary particles with size of about 100 nm, each of which takes on a flake-like appearance. After calcination, themorphology and structure changed completely, resulting in an irregular shape of the primary particles with size of about 70~300 nm (Fig. 2c,d). Therefore, the precursor primary particles with smaller size aggregated into larger ones with the size of secondary particles remained almost unchanged during calcination. In order to investigate the composition and chemical state of the atoms of the samples before and after annealing (p-PhyA-LMFP and IC-LMFP), X-ray photoelectron spectroscopy (XPS) was subsequently conducted. As shown in Fig. 3c,d, the C1s spectrum of p-PhyA-LMFP displays three main peaks at 284.60, 286.02 and 288.35 eV which are attributed to the forms of C–C, C–O and C=O, respectively^29^; the O1s spectrum possesses two main peaks at 530.86 and 532.70 eV corresponding to O–C and O–P functional groups, respectively^30,31^. The presence of C–O indicates that the precursor persist its phytate structure after solvothermal reaction. However, the peak corresponding to C–O in C1s spectrum became much weaker and the one corresponding to O–C in O1s almost disappeared after calcining at 750 °C, as shown in Fig. 3e,f, which indicate the break of C–O chemical bond and destruction of the phytate structure. Hence, the phytate precursor finally pyrolyzed into phosphate, and the hexatomic carbon ring of PhyA would transform into internal carbon (IC) flakes, which is further proved by the Raman spectroscopy analysis results shown in Fig. S1. In addition, Fig. S2 display the Fe2p and Mn2p spectra of p-PhyA-LMFP and IC-LMFP, illustrating the presence of Fe(II) and Mn(II) species in the materials.

Preserving the nanostructure of the IC-LMFP derived from PhyA during the whole pyrolysis process, especially at steps involving elevated temperature, is key to producing high quality cathode materials. Fig. S3 shows the TGA and DTG plots of PA-LMFP and PhyA-LMFP. The TGA curves descend under 200 °C, relating to the free water initially released in both phytate and phosphate. After that, two stages of weight loss take place in the phosphate. As for phytate, the multistage weight losses were observed. In order to demonstrate their difference explicitly, the differential of both TGA results are required. Two peaks at about 270 °C and 380 °C appears in the two differential curves, which correspond to the elimination of free water and bound moisture. The subtle variations of the DTGs could be addressed to two different structures. In the range of 430–700 °C, there are two obvious decreases of phytate due to the oxidation of organic component with removal of partial carbon in the form of CO_2_^32,33^. The carbon decomposition can be further verified by the final carbon content (1.6%) of the obtained IC-LMFP, which is far less than the theoretical calculating max value (7.6%). The thermoanalytical studies indicate that phytates could be resolved into phosphates and IC beyond 430 °C. Furthermore, it can also be proved that the phytate is not disrupted during the solvothermal process.

**Figure 4:**
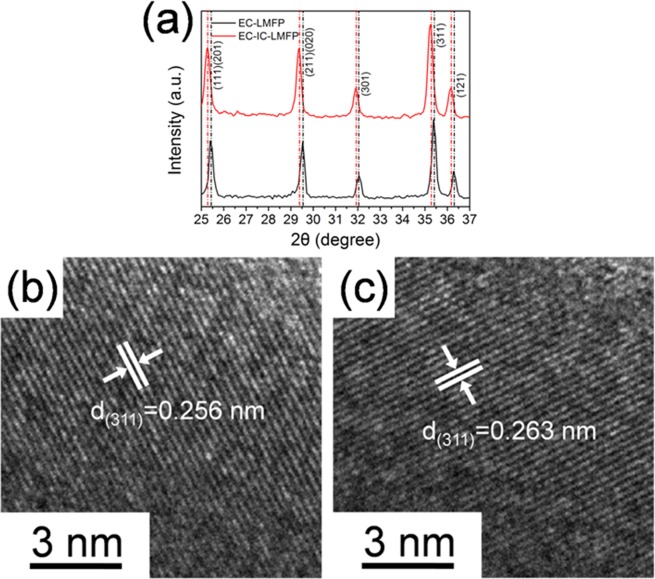
(**a**) Local amplification XRD pattern of EC-LMFP and EC-IC-LMFP; (**b**,**c**) HRTEM image of EC-LMFP and EC-IC-LMFP.

For the sake of investigating the influence of IC on the crystal structure of LMFP, we amplified the XRD patterns of Fig. 1 in the range of 25°~37°, shown in Fig. 4a. It can be observed that the peaks representing the (111), (201), (211), (020), (301), (311) and (121) crystal planes of LMFP all shifted about 0.16° to smaller angle. It is possible to result in the increment of lattice parameters^34^. The value of full width half maximum of (311) plane of EC-IC-LMFP is 0.249°, larger than the 0.231° of EC-LMFP, proving that EC-IC-LMFP is of wider distribution of lattice constants owing to the presence of IC inside the LMFP particles. Additionally, the Williamson-Hall analysis was performed to quantify the amount of strain in the materials, as shown in Fig. S4. The amount of residual strain in EC-IC-LMFP is 0.102, larger than the 0.101 of IC-LMFP and 0.023 of LMFP. The larger residual strain probably results from IC flakes laid within the LMFP crystals. High resolution transmission electron microscopy (HRTEM) was subsequently performed, as shown in Fig. 4b,c. EC-LMFP and EC-IC-LMFP both display highly ordered single crystal structures but with different interplanar spacing. The interplanar spacing of EC-IC-LMFP is 0.263 nm, a little larger than that of EC-LMFP (0.256 nm), in accordance with the local amplification XRD result of Fig. 4a. This larger interplanar spacing probably result from the presence of IC, and could broaden the Li^+^ transport pathways, bringing better electrochemical performances^34^. In comparison, XRD patterns of the precursors using phosphoric acid (PA) or phytic acid (PhyA) as phosphorous source (p-PA-LMFP and p-PhyA-LMFP, respectively) are provided in Fig. S5. It is clear that from the local amplification XRD pattern of Fig. S5b, no obvious shifting can be observed. This is because that IC has not formed after the solvothermal process, thus no change of interplanar spacing occurs.

**Figure 5:**
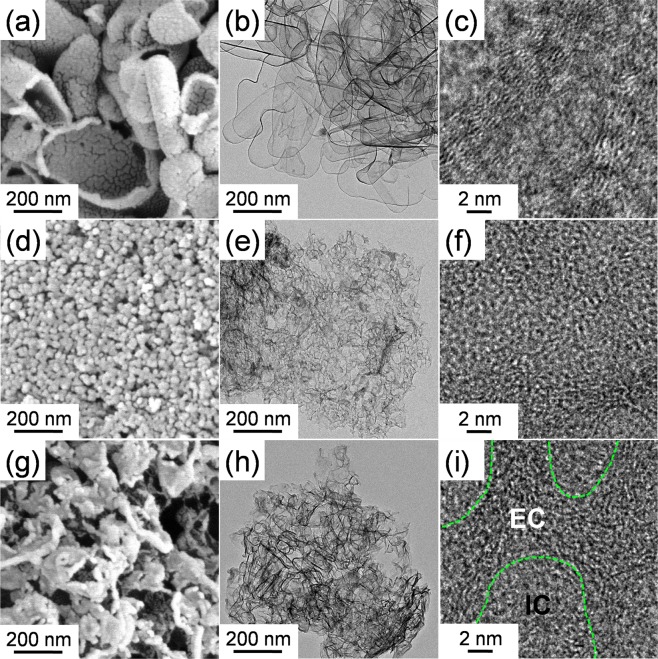
(**a**–**c**) SEM, TEM and HRTEM images of EC; (**d**,**f**) SEM, TEM and HRTEM images of IC; (**g**–**i**) SEM, TEM and HRTEM images of EC-IC.

In order to directly study EC, IC and EC-IC, EC-LMFP, IC-LMFP and EC-IC-LMFP were dissolved in excessive 3 M HCl solution under intense stirring and boil for 5 min to remove the LMFP phase. After cooling down to room temperature, EC, IC and EC-IC were collected by centrifugation and washed for 5 times before dried at 60 °C overnight. The IC in IC-LMFP cannot be obviously seen from SEM image of Fig. 2, for it exist inside the LMFP crystals, but it could be separated and observed. Figure 5 presents the SEM, TEM and HRTEM images of EC, IC and EC-IC after dissolving the LMFP phase. It can be seen from Fig. 5a,b that EC is of hollow capsule shape, which is the result of the removal of LMFP, indicating the good coating of EC on LMFP, while IC exhibits a thin flake structure consisting of lots of tiny particles (Fig. 5d,e). It can be observed from Fig. 5f that IC is amorphous and highly disordered, and there exist many micropores in the IC flake, which is advantageous to the migration of Li^+^. It can be inferred that there exist a number of defects on IC, bringing lots of active sites for oxygenic groups and will probably offer extra capacity. Differently, the HRTEM image of Fig. 5c displays some relatively ordered areas, illustrating the better graphitization of EC than IC. Moreover, as Fig. 5g,h display, EC-IC presents an interlacement of hollow capsules and thin flakes. There exist some highly disordered and relatively ordered areas in Fig. 5i, indicating the coexistence of IC and EC. This coexistence will bring a 3D conductive network with IC providing transport pathways for electron from the internal and EC from the external. XRD analysis was carried out for EC, IC and EC-IC, and the patterns are displayed in Fig. S6a. These three samples all show two broad peaks at approximately 23° and 43° corresponding to (002) and (100) of the pseudographitic domains^35^, respectively. The graphitization degrees of the samples can be compared using an empirical parameter (R), defined as the ratio of height of the (002) Bragg peak to the background^36^. From Fig. S6b, it can be calculated that the R value of EC (3.06) is higher than IC (2.17), indicating the higher degree of graphitization of EC than IC, which agrees with the Raman (Fig. S7) and HRTEM results, while the R value of EC-IC (2.27) is in between. The higher degree of graphitization of EC could bring better conductivity^37^, which is beneficial for the electrochemical performances of EC-IC-LMFP.

Nitrogen adsorption-desorption isotherms of EC, IC and EC-IC are schematically shown in Fig. S8a. All samples exhibit a type IV N_2_-sorption isotherms curve with a capillary condensation step (P/P_0_ ≈ 0.9) in the sorption branch, which is a typical characteristic of mesoporous materials^38–40^. The specific surface area of EC-IC is calculated to be 971.1 m^2^ g^−1^, which is higher than those of IC (499.8 m^2^ g^−1^) and EC (578.3 m^2^ g^−1^). The corresponding total pore volumes are 2.38, 2.10 and 1.23 cm^3^ g^−1^, respectively. This chemical-etching process generates mesopores with a pore size distribution concentrated at a range from 3 nm to 15 nm. (see Fig. S8b–d). A sharp peak of EC-IC located at 4 nm, which confirms that the carbon composite combined IC with EC forms plenty of smaller cavity structures compared to those of EC. This further demonstrates the obtained LMFP particles from PhyA are smaller than that from PA. The fine composite structure of 3D carbon framework greatly limits the growth of LMFP during calcination and contributes large electrode-electrolyte interface during electrode reaction. Therefore, the wrapped LMFP nanoparticles will benefit the improvement of electrochemical performance. The XPS results for IC and EC are shown in Fig. S9. The peaks of C1s centered at 286.44 eV, 288.06 eV and 289.02 eV are ascribed to C–O, C=O and O=C–O, respectively, suggesting that a number of oxygenic functional groups decorated the surface and edges of IC. The existence of oxygenic groups is beneficial to the enhancement of energy density^41^.

**Figure 6:**
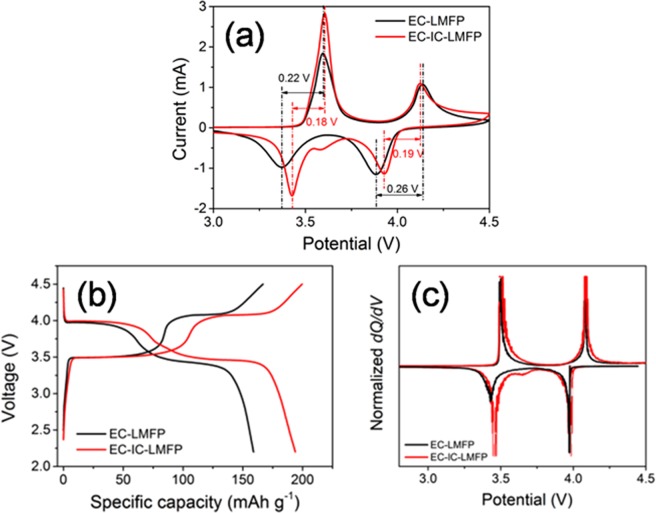
(**a**) Local amplification of CV curves of EC-LMFP and EC-IC-LMFP; (**b**) Galvanostatic charge/discharge curves of EC-LMFP and EC-IC LMFP at the rate of 0.1 C (1 C = 170 mA g^−1^) and (**c**) corresponding differential curves.

For the sake of investigating the electrochemical performances of the materials, cyclic voltammetry (CV) test was conducted and the results are shown in Fig. S10. Two pairs of anodic/cathodic peaks are presented in the CV curve of EC-LMFP. The anodic peaks at 3.59 and 4.14 V represent the oxidation of Fe(II) to Fe(III) and Mn(II) to Mn(III), and the cathodic peaks at 3.37 and 3.88 V are attributed to the reduction of Fe(III) to Fe(II) and Mn(III) to Mn(II), which agree with the redox reaction of LMFP, while the CV curve of EC-IC-LMFP displays an additional cathodic peak at about 3.58 V. Meanwhile, the cathodic peaks corresponding to the reduction of Fe(III) to Fe(II) and Mn(III) to Mn(II) of EC-IC-LMFP shifted to the right (3.43 and 3.93 V, respectively). Generally, there must be an anodic peak corresponding to the extra cathodic peak. The absence of this anodic peak is probably owing to the similar peak position to the much stronger one of Fe(II) to Fe(III). The CV area of EC-IC-LMFP is larger than that of EC-LMFP, demonstrating a higher capacity of EC-IC-LMFP than EC-LMFP. In addition, the potential separations of the two pairs of main anodic/cathodic peaks of EC-IC-LMFP are 0.18 and 0.19 V, lower than those of EC-LMFP (0.22 and 0.26 V), as shown in Fig. 6a. Therefore, it can be illustrated that EC-IC-LMFP possesses higher ionic conductivity owing to the existence of IC. Moreover, the extra anodic/cathodic peaks are associated with the additional capacity, and it is further proved by the derivative of galvanostatic charge/discharge (GCD) results displayed in Fig. 6c.

A comparison of the GCD curves of EC-IC-LMFP and EC-LMFP was made subsequently, as presented in Fig. 6b. It is obvious that EC-IC-LMFP displays an additional discharge voltage plateau at about 3.6 V, bringing extra discharge capacity, possessing 193 mAh g^−1^ at 0.1 C (1 C = 170 mA g^−1^), exceeding the theoretical capacity of LMFP (170 mAh g^−1^). Based on this, the mass energy density of EC-IC-LMFP is calculated to be 698 Wh kg^−1^, larger than that of EC-LMFP (567 Wh kg^−1^), which is consistent with the constant resistant discharge (CRD) test results shown in Fig. S12. Figure 6c shows the differential curve of GCD diagram of EC-IC-LMFP, where three pairs of peaks can be observed, demonstrating that the exceeded capacity is ascribed to the contribution of other sources besides that of LMFP. This is in accordance with the corresponding CV results in Fig. 6a. The exceeding of theoretical capacity of EC-IC-LMFP is mainly ascribed to the IC flakes. As discussed in the above, the IC flakes are rich in oxygenic groups (–C=O, –COOH, etc.), and the presence of surface oxygenic groups in carbonaceous materials can bring enhancement of energy density^42,43^. These surface oxygenic groups will bring about Faradaic reactions, with the corresponding redox peaks locating at about 3.0 V and 3.4 V vs. Li^41^. These peaks are principally associated with the reaction: –C = O + Li^+^  + e^−^ ↔ –C–OLi^44,45^.

**Figure 7:**
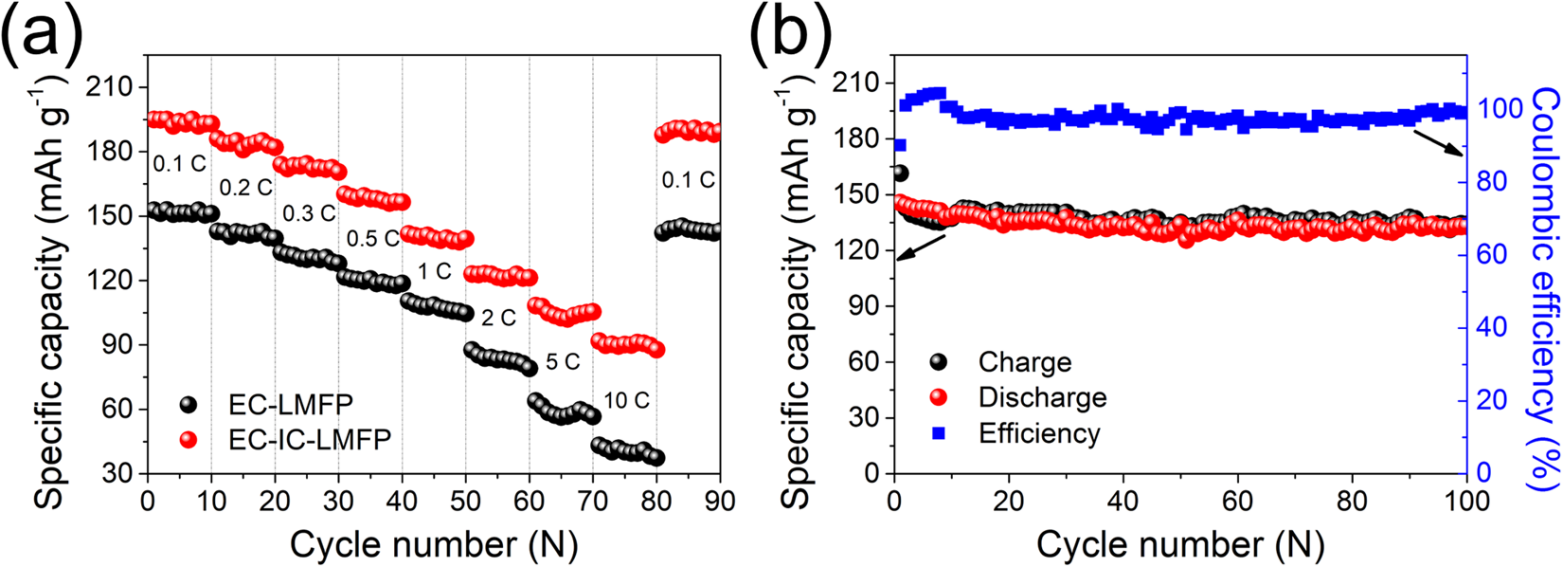
(**a**) Rate capability of EC-LMFP and EC-IC-LMFP; (**b**) cycling performance of EC-IC-LMFP with corresponding Coulombic efficiency.

**Figure 8:**
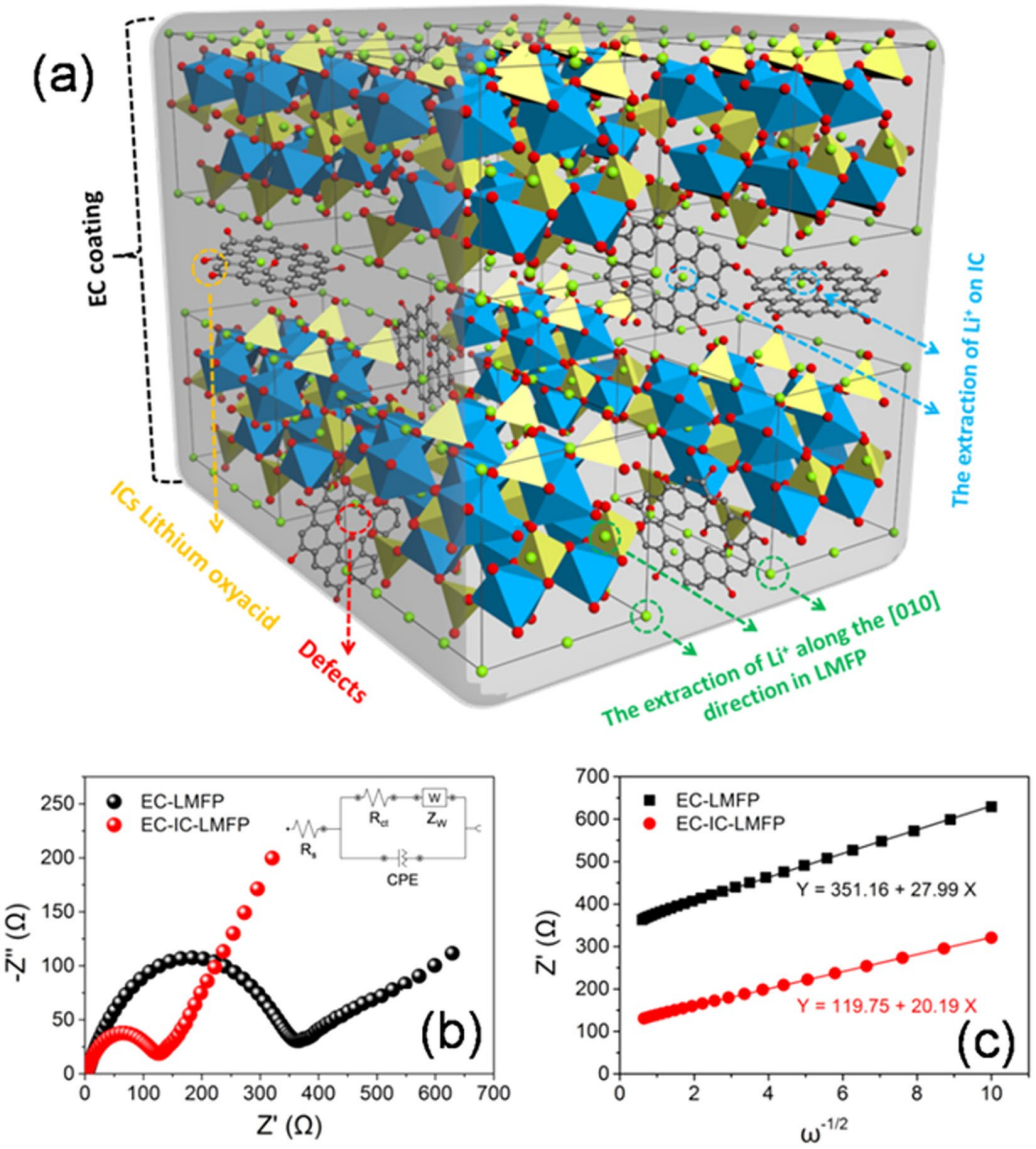
(**a**) The anatomical structure diagrammatic sketch of EC-IC-LMFP during charging process; (**b**) Nyquist plots of EC-LMFP and EC-IC-LMFP after 5 charge/discharge cycles (the inset presents the equivalent circuit established for the simulation of the Nyquist plots); (**c**) variations and fittings between Z’ and the reciprocal square root of the angular frequency (ω^−1/2^) in the low-frequency region for EC-LMFP and EC-IC-LMFP.

The additional capacity is probably relevant to the excess of Li^+^. For the sake of proving this speculation, ICP-AES analysis was carried out for EC-LMFP and EC-IC-LMFP to obtain the molar ratio of Li:Mn:Fe:P elements after charging, and the results are listed in Table S2. Compared to the ICP-AES results of the electrode before charging, after being charged, the molar ratio of Li:Mn:Fe:P is 0.07:0.50:0.54:1 for EC-LMFP, about 93% of Li transferred to the anode, and most of the transferred Li^+^ could return to cathode after discharging. However, the actual amount of Li by using PhyA as phosphorous source is obviously more than that utilizing PA, reaching 1.29:0.50:0.51:1, which indicates the excess amount of Li. After charging, the molar ratio of Li:Mn:Fe:P turns 0.15:0.49:0.52:1 for EC-IC-LMFP. More lithium ions are able to transfer to anode after charging for EC-IC-LMFP than EC-LMFP, most of which will be back to cathode reversibly during discharging. Some reports demonstrate that the extra Li can bring about reversible redox reaction. The additional Li can bind with the oxygenic groups or defects on IC flakes, undergoing reversible Faradaic reactions, which is an explanation of the excess capacity. Rate capabilities of EC-IC-LMFP and EC-LMFP were evaluated by testing the electrodes at rates of 0.1, 0.2, 0.3, 0.5, 1, 2, 5, 10 and 0.1 C with 10 charge-discharge cycles per rate, as shown in Fig. 7a. Obviously, the EC-IC-LMFP possesses a better rate performance and higher reversible capacity than EC-LMFP. Even at the rate of 10 C, it still retains a reversible specific capacity of 90 mAh g^−1^. After changing the rate back to 0.1 V, EC-IC-LMFP can reach an almost complete capacity recovery, suggesting good stability during cycling. Fig. 7b presents the long-term cycling performance along with the corresponding Coulombic efficiency of EC-IC-LMFP at the rate of 1 C, from which it can be seen that the capacity retention is 93% even after 100th cycle, and the Coulombic efficiency stays over 90% during the whole process. To illustrate the charge process of EC-IC-LMFP, a diagrammatic sketch is made and displays as Fig. 8a. The IC flakes exist inside LMFP crystal, leading to the increase of interplanar spacing. The red spheres on IC represent oxygen atoms, forming oxygenic groups on the surface of IC flakes, which are able to capture and release Li ions during discharging and charging. The extra Li^+^ can be stored reversibly between IC flakes or in the defects at the edge sites. When charging, most of Li^+^ are extracted from the LMFP lattice according to the common way shown in green dotted lines, while the excessive part of Li^+^ can deintercalate from the IC flakes, as the blue dotted lines displays. The IC flakes can not only enhance the reversible capacity, but serve to improve ionic conductivity, which can be proved from the EIS results shown in Fig. 8b,c.

The lithium diffusion coefficients (D) for EC-IC-LMFP and EC-LMFP can be calculated following the equation: D = *R*^2^*T*^2^/2*A*^2^*n*^4^*F*^4^*C*^2^*σ*^2^, where R, T, A, n, F, C and σ stand for the gas constant, temperature, cathode disk area, number of moles of electrons transferred corresponding to the oxidation of cathode material per mole, Faraday constant, Li ion concentration, and Warburg factor associated with Z′ (Z′ ∝ σω^−1/2^), respectively. The Warburg factor σ of EC-IC-LMFP and EC-LMFP can be obtained from the linear fitting of Z′-ω^−1/2^ plots (Fig. 8c), where ω is the angular frequency, then D values were calculated according to the equation mentioned above (3.23 × 10^−13^ for EC-LMFP and 6.20 × 10^−13^ cm^2^ s^−1^ for EC-IC-LMFP). Obviously, the D value of EC-IC-LMFP is larger than that of EC-LMFP, demonstrating that the presence of IC benefits the diffusion of Li. When measuring by four-point probe resistivity tester, the typical resistivity of EC-LMFP is tested to be about 123 Ω cm, while that of EC-IC-LMFP is about 54 Ω cm. Additionally, the electronic conductivity of EC-IC-LMFP is higher than that of EC-LMFP, which is illustrated in the corresponding EIS spectra (Fig. 8b). Furthermore, the lithiation mechanism was studied by galvanostatic intermittent titration (GITT) measurements to evaluate the kinetic properties^46^ (Fig. S13). GITT mode was applied with 30 min continuous charge/discharge at 0.05 mA cm^−2^ followed by 30 min rest intervals. The electrochemical process drove the phase transformation during charge/discharge process of LMFP with different average cell volumes^47^. On charging process, the increasing of the plateau from 3.5 V to 4.1 V causes little additional change of lithium diffusivity, which faded from ~10^−11^ to ~10^−12^ cm^2^ s^−1^ as cell volume decreased. The diffusion coefficients in the Mn redox regime are consistently lower than those in the Fe redox regime for there is a large resistance for Li^+^ to pass through the two-phase interface^15,48^. The Li-ion diffusivity in EC-IC-LMFP displays that diffusion associated with the plateau potentials is much lower than that with the slope areas, suggesting that initial lithiation takes place on accessible sites in EC-IC-LMFP. As all the sites are gradually lithiated, Li^+^ will then transfer inside the crystals. When it turned to discharge, the phenomenon shows a high conformity. The formulas used to calculate the bulk diffusivity are introduced in Supplementary Information.

Significantly, the larger mass energy density of EC-IC-LMFP than LCO (~520 Wh kg^−1^) makes up for the drawback of LMFP in volumetric energy density. The compact density of EC-IC-LMFP and EC-LMFP were tested to be both 2.3 g mL^−1^, same as the commercial LFP, and the volumetric energy density of EC-IC-LMFP is estimated to be 1605 Wh L^−1^. It can be estimated that if the EC-IC-LMFP is employed as cathode material for a LIB, the size of the full battery is 28% smaller than commercial LFP battery and only 24% larger than the commercial LCO battery, which is acceptable considering the longer lifetime, higher safety, much lower cost and much better benignity to environment of EC-IC-LMFP, while for commercial LFP, it will be 68% larger than commercial LCO battery.

## Conclusions

A composite of LiMn_0.5_Fe_0.5_PO_4_, external carbon coating and internal embedded carbon flakes, EC-IC-LMFP, was prepared by employing phytic acid as phosphorus source *via* solvothermal procedure followed by calcination. The precursor is globular phytate secondary particles composed of tablet-shaped primary nanoparticles. It is proved that the introduction of a small amount of IC could improve the ionic conductivity of LMFP, and meanwhile enhance the reversible capacity. EC-IC-LMFP possesses better electrochemical performances than EC-LMFP prepared using PA, presenting a very large specific capacity of 193 mA h g^−1^ at the rate of 0.1 C, exceeding the theoretical one of LMFP. The Faradaic reaction between Li^+^ and oxygenic groups at defects on IC is the reason for the excess capacity. It needs to be emphasized that the very high discharge capacity of EC-IC-LMFP combined with a high compact density could bring a high volumetric energy density, estimated to reach 1605 Wh L^−1^. Its inherited safety feature can prohibit these materials from oxygen release, making it possible to relieve the cell venting and swelling, reducing the risk of fire or explosion, and simplifying battery management system. Together with the eco-friendliness, long cycling life and low cost, it will be a promising candidate to batteries for electrical vehicles and is possible to be an optional in the batteries for portable devices.

## Methods

### Preparation of IC-LMFP

All chemical reagents, including PhyA (aqueous solution, 50 wt.%), H_3_PO_4_ (aqueous solution, 85 wt.%) MnSO_4_·H_2_O, FeSO_4_·7H_2_O and LiOH·H_2_O were of analytical grade (Keshi Chemical Reagent Co., Ltd.) and used without any further purification. The molar ratio of PhyA:MnSO_4_:FeSO_4_:LiOH was 1:3:3:18 and the total number of moles were 0.065. A mixture of the PhyA,MnSO_4_·H_2_O and FeSO_4_·7H_2_Owas introduced into a glass beaker with 70 mL deionized (DI) water and stirred for 30 min to make a homogeneous solution. Followed by adding 150 mL ethylene glycol (EG), the system formed a yellowish-brown transparent solution. Then 80 mL as-prepared LiOH·H_2_O aqueous solution was added dropwise under an argon flow. After bubbling for 0.5 h, the pH of the suspension system was adjusted to 7.2. Later on, the whole suspension was immediately transferred to an autoclave under continuous magnetic stirring. The successive thermal treatment was carried out at 200 °C for 2 h, and then cooled down to ambient temperature.

The sediment was separated from the suspension by centrifugation at 4000 rpm and washed with ethanol once. The obtained precursor (p-PhyA-LMFP) was vacuum dried at 80 °C overnight. The obtained powder was subsequently pressed into tablets at 150 kg cm^−2^. The crucible with tablets was inserted into a tubular furnace under a high-purity argon atmosphere followed by sintering at 750 °C for 2 h. Finally, the fine IC-LMFP powder was obtained by grinding.

### Preparation of EC-IC-LMFP

To further improve the conductivities of the products, the EC was introduced by carbonization of glucose. In a typical preparation, ball milling was used to fully mix and grind the p-PhyA-LMFP and glucose. The glucose-to-precursor weight ratio was 1:10. The final EC-IC-LMFP sample was synthesized as the above calcination method.

### Preparation of LMFP and EC-LMFP

LMFP and EC-LMFP were synthesised through the same approach except that phosphoric acid (PA) was employed instead of PhyA as phosphorus source.

### General characterizations

A JEOL JSM-7500F scanning electron microscope (Tokyo, Japan) was applied to obtain the field emission scanning electron microscopy (FESEM) images. Surface morphology and interplanar spacings of the samples was investigated from transmission electron microscopy (TEM) and high-resolution transmission electron microscopy (HRTEM) images obtained using a Zeiss Libra 200FE transmission electron microscope (Oberkochen, Germany) operating at an accelerating voltage of 200 kV. The specific surface areas were taken on Micromeritics Qunatachrome Nova 2000e automatic surface area analyzer (Boyton Beach, USA) at 77 K using the Brunauer-Emmett-Teller method, while pore size distributions (PSDs) were calculated according to the density functional theory (DFT) method from the adsorption branches of the isotherms. The X-ray diffraction (XRD) data were recorded on a Haoyuan DX-2800X-ray diffractometer (Dandong, China) equipped with Cu Kα radiation. X-ray photoelectron spectroscopy (XPS) results were obtained using a Thermo Fisher Scientific ESCALAB250Xi spectrometer (Maple Plain, USA) with a MgKα X-ray (1253.6 eV) excitation source running at 15 kV. A Renishaw inVia spectrometer (Wotton-under-Edge, UK) with excitation laser at 532 nm was employed to record Raman spectra. The thermogravimetric analysis (TGA) was performed on a Netzsch STA 449 F5 Jupiter thermal analyzer (Selb, Germany) under a heating rate of 10 °C min^−1^ in a high-purity argon atmosphere from room temperature to 750 °C. Elemental analysis data were collected using a EuroEA3000 Analyzer (Leeman, USA). Inductively coupled plasma-atomic emission spectroscopy (ICP-AES) were conducted on a ThermoElemental IRIS Advantage atomic emission spectrometer (Waltham, USA) for the accurate measurement of the elements.

### Electrochemical tests

The discharge/charge test and cycling performance were recorded at room temperature using a Newware CT-4008 battery testing system (Shenzhen, China). The cathodes were prepared by mixing active materials, carbon black as conductive agent and polyvinylidene fluoride (PVDF) as binder in a weight ratio of 8:1:1 in appropriate amount of N-methylpyrrolidone (NMP) to form a homogeneous slurry. The dispersed slurry was cast onto an Al foil current collector and dried at 100 °C for 2 h. All the electrodes were pressed and cut into circular disks of 14 mm in diameter with an active material loading of about 1~2 mg cm^−2^. The coin-type cells CR2032 were assembled with the test electrodes, Li metal counter electrodes, a polymer membrane separator (Celgard, 2400) and 1 M LiPF_6_ dissolved in a 1:1 volume ratio of ethylene carbonate (EC): dimethyl carbonate (DMC) in an argon-filled glove box (H_2_O < 2 ppm, O_2_< 0.5 ppm, Dellix, China). Cyclic voltammetry (CV) data were collected on a Metrohm Autolab PGSTAT 302 electrochemical workstation (Utrecht, The Netherlands) at a scan rate of 0.1 mV s^−1^ in the voltage range of 0~3.0 V. Electrochemical impedance spectroscopy (EIS) measurements were performed in the frequency range of 0.01~100 kHz with an applied amplitude of 5 mV at a charged stage.

## Supplementary information

Supplementary Information
