# Supplementary material for: A phytic acid derived LiMn0.5Fe0.5PO4/Carbon composite of high energy density for lithium rechargeable batteries
Source: Sci Rep. 2019 Apr 30;9:6665. doi: 10.1038/s41598-019-43140-7 (PMC6491431; doi:10.1038/s41598-019-43140-7)
Supplement: Supplementary file 1 — Supplementary Information [file 41598_2019_43140_MOESM1_ESM.docx]

**Supplementary Information**

**A Phytic acid derived LiMn_0.5_Fe_0.5_PO_4_/Carbon composite of high energy density for lithium rechargeable batteries**

Yan Meng^1+^, Yujue Wang^2+^, Zhaokun Zhang^3^, Xiaojuan Chen^1^, Yong Guo*^23^ and Dan Xiao*^123^

^1^ *School of Chemical Engineering, Sichuan University, Chengdu 610065, China*

^2^ *Institute of New Energy and Low-Carbon Technology (INELT), Sichuan University, Chengdu 610207, China*

^3^ *College of Chemistry, Sichuan University, Chengdu 610064, China*

^+^ These authors contributed equally.

*These are co-corresponding authors.

Table S1 The contents of Li, Mn, Fe and P measured by ICP-AES analysis for EC-LMFP and EC-IC-LMFP (the unit is ppm)

| Samples | Li | Mn | Fe | P |
| --- | --- | --- | --- | --- |
| EC-LMFP | 0.5305 | 1.983 | 2.184 | 2.281 |
| EC-IC-LMFP | 0.5941 | 1.810 | 1.868 | 2.036 |


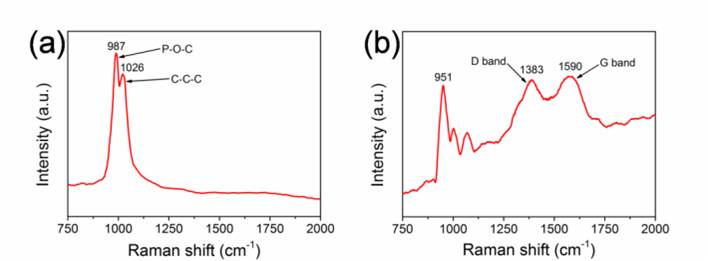


Fig. S1. Raman spectra of (a) p-PhyA-LMFP and (b) IC-LMFP


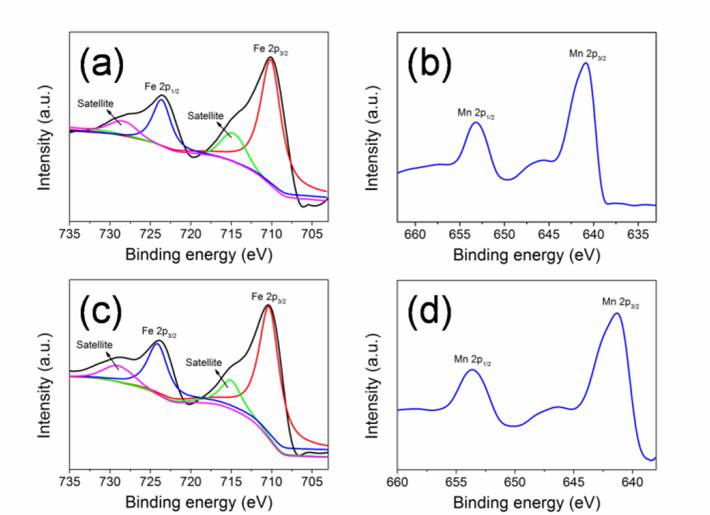


Fig. S2. (a and b) XPS spectra of p-PhyA-LMFP: (a) Fe2p, (b) Mn2p; (c and d) XPS spectra of IC-LMFP: (c) Fe2p, (d) Mn2p


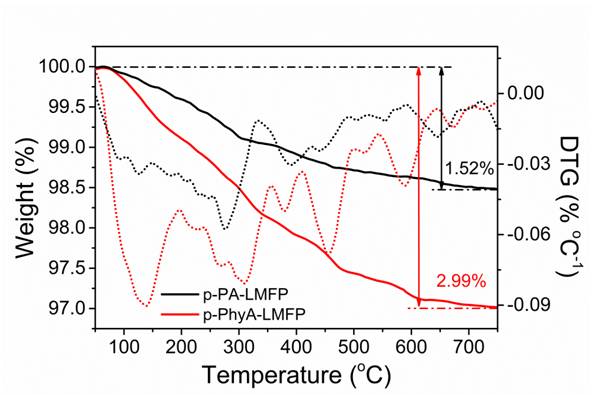


Fig. S3. TG and DTG curves of p-PA-LMFP and p-PhyA-LMFP


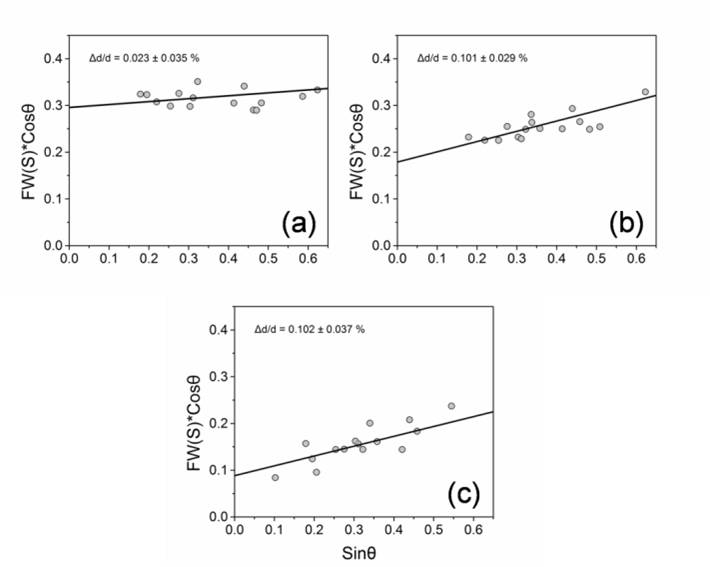


Fig. S4. Williamson-Hall plots of (a) LMFP, (b) IC-LMFP and (c) EC-IC-LMFP


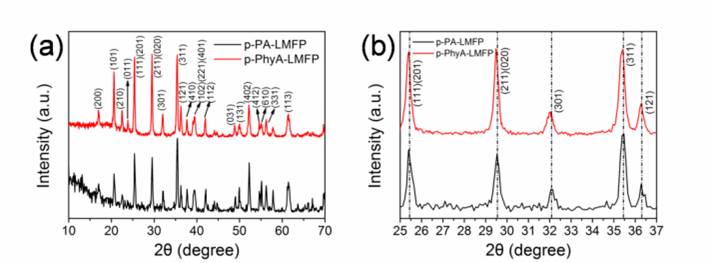


Fig. S5. (a) XRD and (b) local amplification XRD pattern of p-PA-LMFP and p-PhyA-LMFP


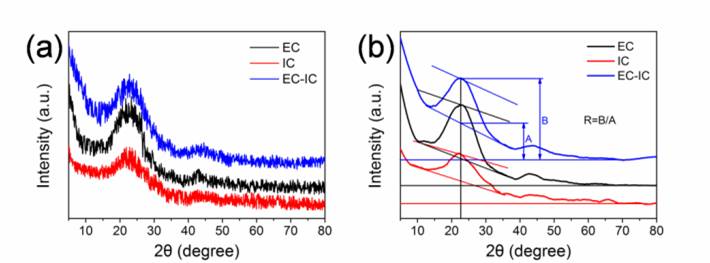


Fig. S6. (a) XRD and (b) smoothed XRD patterns of EC, IC and EC-IC


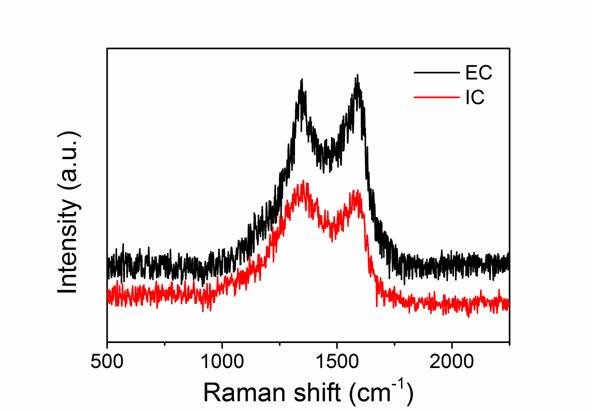


Fig. S7. Raman spectra of EC and IC


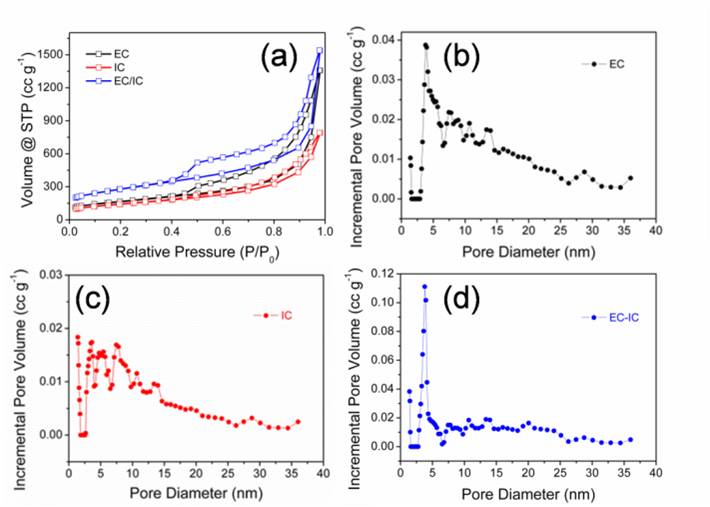


Fig. S8 (a) Nitrogen adsorption-desorption isotherms of EC, IC and EC-IC; (b-d) pore size distributions of EC, IC and EC-IC calculated according to the DFT method from the adsorption branches of the isotherms


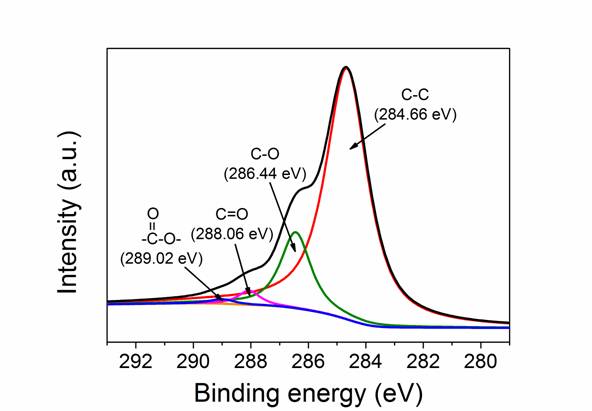


Fig. S9. C1s XPS spectrum of IC


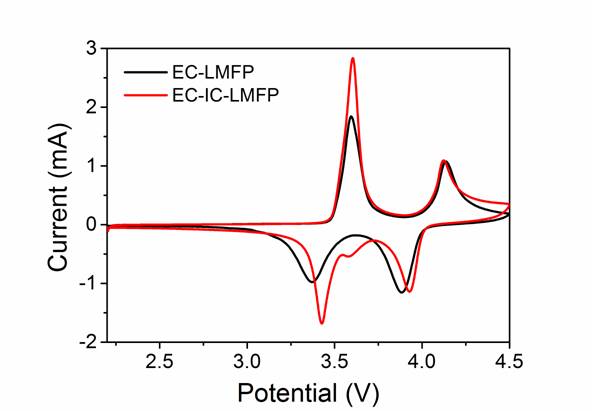


Fig. S10. CV curves of EC-LMFP and EC-IC-LMFP at a scan rate of 0.1 mV s^-1^


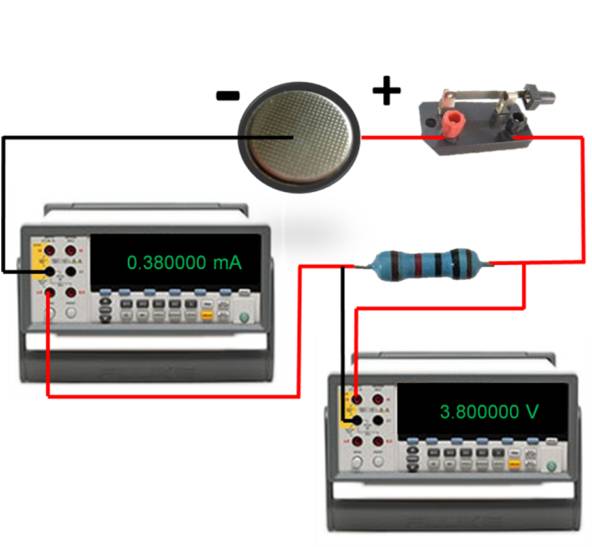


Fig. S11. Illustration of the operating circuit through the constant resistance discharge (CRD) process


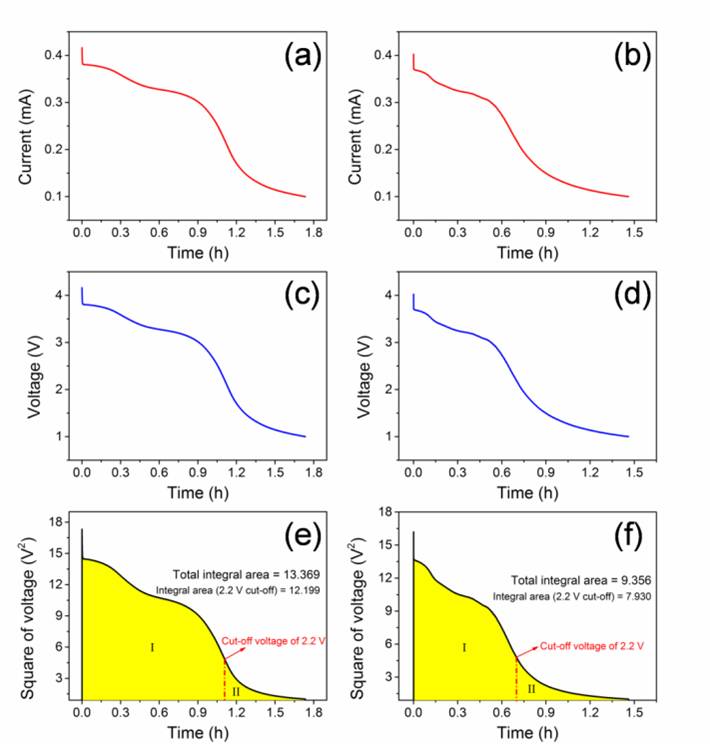


Fig. S12 (a and b) The curves of current versus time for EC-IC-LMFP (a) and EC-LMFP (b); (c and d) the curve of voltage versus time for EC-IC-LMFP (c) and EC-LMFP (d) during the constant resistance discharge (CRD) process; (e and f) the curves of square of voltage versus time for EC-IC-LMFP (e) and EC-LMFP (f)during the CRD process and the corresponding integral areas

In a typical battery test, a galvanostatic discharge is usually conducted. In order to better understand the performance and output power of the electrodes, instead of controlling the current to be constant, the constant resistance discharge (CRD) method has been applied. A load resistor (*R*_load_) of 10 kΩ is utilized. The output voltages and currents were recorded on two Fluke 8846A digital multimeters (Everett, USA) during discharging process according to the circuit shown in Figure S11, and the results for EC-IC-LMFP and EC-LMFP are presented in Fig. S12. The advantage of this technique is that it could achieve rather high power values, depending on the internal resistance *R*_internal_ of the battery tested. The current is very large at the beginning, but keeps decreasing in the whole discharging process, as Fig. S12a and b display. The voltage exhibits the same variation (Fig. S12c and d), for the reason that the resistance of external circuit is constant. Furthermore, both EC-IC-LMFP and EC-LMFP display two voltage plateaus at approximately 3.8 and 3.3 V. From the results, the energy densities (*E*) can be calculated (*E* = $\int_{\text{0}}^{\text{t}} \text{UI·d}t$). For the spontaneous discharging process with a constant resistance of external circuit, E = $\frac{\text{1}}{\text{R}}\int_{\text{0}}^{\text{t}} \text{U}^{\text{2}}\text{·d}\text{t}$. Fig. S12c and d are provided with time (t) as X axis, square of voltage (U^2^) as Y axis. The integral areas of Fig. S12c and d represent the integral term in the above equation. The value of mass energy densities for EC-IC-LMFP and EC-LMFP were calculated to be 711 Wh kg^-1^ and 557 Wh kg^-1^, respectively. The integral area (I) in Fig. S12e and f times 1/R represents the energy outputted when the spontaneous discharging process stops at 2.2 V, the same cut-off voltage as the galvanostatic discharging process. Under this condition, the mass energy density for EC-IC-LMFP and EC-LMFP were calculated to be 649 and 472 Wh kg^-1^, respectively. It can be concluded that the mass energy density of EC-IC-LMFP is higher than EC-LMFP even through the spontaneous discharging process, which is consistent with the result involving galvanostatic discharging process, indicating a better performance of output power for EC-IC-LMFP than EC-LMFP. In addition, it can be calculated from Fig. S12e and f that the proportion of outputted energy of EC-IC-LMFP within the range of 1.0 ~ 2.2 V is 8.8% of the total energy, less than the 15.2% of EC-LMFP. This proportion of outputted energy below 2.2 V is ascribed to the trapped zones of LMFP resulted from its poor conductivity. So it can be inferred that the trapped zones of LMFP in EC-IC-LMFP is smaller than that of EC-LMFP, illustrating the positive role IC plays in electrochemical performances.

Table S2 The contents of Li, Mn, Fe and P measured by ICP-AES analysis for EC-LMFP and EC-IC-LMFP after charging (the unit is ppm)

| Samples | Li | Mn | Fe | P |
| --- | --- | --- | --- | --- |
| EC-LMFP | 0.03376 | 1.985 | 2.084 | 2.136 |
| EC-IC-LMFP | 0.06866 | 1.762 | 1.904 | 2.027 |

Galvanostatic intermittent titration (GITT) was first developed by Weppner and Huggins to establish a following relation between long duration voltage transients and bulk diffusivity.

$\tilde{D}= {\frac{4}{\pi}\left( \frac{m_{b}V_{M}}{M_{B}S} \right)}^{2}\left( \frac{\Delta E_{S}}{\tau(\frac{dE}{d\sqrt{t}})} \right)^{2}\left( t \ll\frac{L^{2}}{D} \right)$ (1)

When E vs. $\sqrt{t}$ exhibits straight-line behavior during the duration of the current pulse, this equation can be simplified into:

$\tilde{D}= {\frac{4}{\pi\tau}\left( \frac{m_{b}V_{M}}{M_{B}S} \right)}^{2}\left( \frac{\Delta E_{S}}{\Delta E_{t}} \right)^{2}\left( t \ll\frac{L^{2}}{D} \right)$ (2)

Where *τ* is current pulse time (sec), *m_B_* is the active material mass (g), *M_B_* is the atomic weight of the active material (g mol^-1^), *V_M_* is the molar volume of the sample (cm^3^ mol^-1^), *S* is the contact surface area (cm^2^).


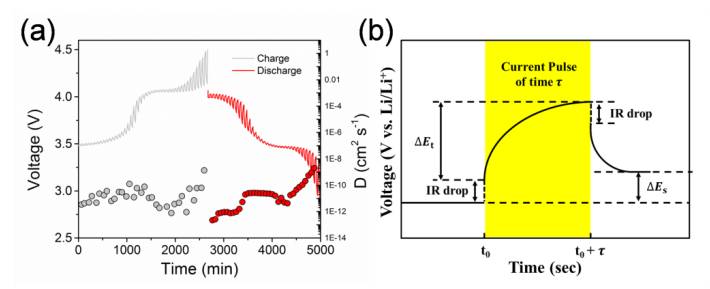


Fig. S13. (a) OCV measured for EC-IC-LMFP. Intermittent discharge mode was used with intervals of 30 min continuous discharge at 0.05 mA cm^-2^ followed by 30 min rest intervals; (b) schematic of GITT technique
